# The association between the body roundness index and the risk of chronic kidney disease in US adults

**DOI:** 10.3389/fmed.2024.1495935

**Published:** 2024-12-18

**Authors:** Jiaying Zhang, Xiaofeng Yu

**Affiliations:** ^1^Department of Nephrology, The Third Hospital of Mianyang/Sichuan Mental Health Center, Mianyang, Sichuan, China; ^2^Department of Cardiology, The Third Hospital of Mianyang/Sichuan Mental Health Center, Mianyang, Sichuan, China

**Keywords:** body roundness index, chronic kidney disease, NHANES, obesity, nephrology

## Abstract

**Aim:**

We aimed to systematically assess whether the level of body roundness index (BRI) is associated with the risk of developing chronic kidney disease (CKD) in US adults.

**Methods:**

The studied data was extracted from the National Health and Nutrition Examination Survey (NHANES) spanning from 1999 to 2018. A total of 29,062 participants aged ≥20 years with complete information about BRI and CKD were included in this study. Logistic regression analysis, multivariate linear regression analysis, restricted cubic spline (RCS) plots curve, stratified analysis and receiver operating characteristic (ROC) curve were performed to investigate the association between BRI and CKD.

**Results:**

A total of 29,062 patients were included, involving 4,623 individuals with CKD and 24,439 individuals without CKD. A higher BRI level was substantially related to an increased prevalence of CKD in US adults. After adjusting for confounding variables, the BRI in the fourth quartile was correlated to a higher CKD prevalence (OR: 1.36; 95% CI: 1.10–1.70) compared to the lowest quartile. After adjusting for confounding variables, the BRI in the fourth quartile was correlated to a higher CKD prevalence (OR: 1.36; 95% CI: 1.10–1.70) compared to the lowest quartile. However, in the subgroup analysis stratified by race and body mass index (BMI), no significant associations between BRI and CKD were observed among Mexican participants (OR: 1.10; 95% CI: 0.98–1.23) and those with underweight or normal weight (OR: 0.95; 95% CI: 0.81–1.05). Moreover, a non-linear relationship was found between BRI and the prevalence of CKD. In ROC analysis, BRI demonstrated higher discriminating for CKD (area under the curve: 0.6247; 95% CI: 0.6161–0.6333; optimal cutoff value: 5.161) compared with other indices.

**Conclusion:**

In summary, BRI was independently associated with a higher prevalence of CKD in overweight and obese US adults, excluding Mexican. This may be an important therapeutic target and predictor of CKD. Physicians should advise patients with high BRI scores, especially overweight and obese patients, to embrace healthy lifestyle changes, such as maintaining a balanced diet and engaging in regular physical activity. These changes can help them control their body weight and reduce abdominal fat, ultimately lowering the risk of CKD.

## 1 Introduction

Chronic kidney disease (CKD) is a broad term for a variety of disorders that impair the structure and function of the kidneys (1). It has a significant impact on society's health and finances due to its high incidence rate, high mortality, high disease burden, and combination with other common major chronic diseases (2, 3). The Centers for Disease Control and Prevention in the United States estimate the prevalence of CKD among adults in the United States to be around 15%, with up to 90% of adult CKD patients unaware of their illness (4). The US Department of Health and Human Services Healthy People 2020 initiative aims to reduce CKD prevalence by 10% in the US population.

Obesity is a chronic metabolic disease caused by excessive fat accumulation due to energy intake exceeds consumption in the body. Obesity is a representative risk factor that may contribute to the rising prevalence and progression of CKD to end-stage renal disease (ESRD) (5–7). The specific reasons are as follows: Firstly, obesity-induced hypertension and hyperfiltration are primary factors contributing to the onset of CKD (8–10). Secondly, insulin resistance upregulates the gene expression of pro-fibrotic and pro-inflammatory factors, increasing the risk of CKD progression (11, 12). Thirdly, obesity-induced lipid overload can cause glomerular and tubulointerstitial damage, further exacerbating kidney injury (13–15). In summary, hemodynamic abnormalities, metabolic disorders, lipid toxicity, and inflammatory response collectively contribute to the development and progression of CKD in obese patients.

The most commonly used measurement for obesity is Body Mass Index (BMI), defined by the World Health Organization as overweight at 25 and above, and obese at 30 and above, with Obesity Class I being a BMI of 30–34.9, Class II as 35–39.9, and Class III as 40 or greater (16). However, for Asians, the BMI classification for obese differs slightly, with Obesity Class I defined as a BMI of 27.5–32.5 and Class II as a BMI of 32.5 or greater (17). However, BMI is limited in measuring abdominal obesity since it solely depends on height and body weight and cannot discriminate the ratio of adipose tissue and muscular tissue (18). Therefore, BMI is incapable of distinguishing between obesity types. In 2013, Thomas et al. (19) developed the Body Roundness Index (BRI), which combines height and waist circumference (WC) to predict the percentage of body fat and reflects the proportion of visceral adipose tissue (VAT) for evaluating abdominal obesity. BRI addresses the limitations of conventional indicators like BMI and WC. Elevated BRI has been linked to a higher incidence of hypertension, diabetes, hyperuricemia, cardiovascular mortality (20–23). According to Zhang et al.'s (24) research, in Chinese communities, low eGFR is positively correlated with BRI, which has the potential to be used as an effective indicator for screening kidney disease. However, no studies have been conducted to determine whether BRI is associated with the prevalence of CKD.

Therefore, this study aimed to explore whether the level of body roundness index (BRI) is associated with the risk of developing chronic kidney disease (CKD) in US adults.

## 2 Methods

### 2.1 Study participants

The data analyzed in this study was collected from the National Health and Nutrition Examination Survey (NHANES) from 1999 to 2018. NHANES is a program combining interviews and physical examinations to assess the health and nutritional status of the U.S. population. Participants were selected through a multistage probability sampling to be representative of the entire U.S. population. The interviews were conducted at the participants' homes. The examinations were performed at the mobile center. The public and deidentified information of participants was collected. The survey's design, methods, and data were publicly accessible via its website (http://www.cdc.gov/nchs/nhanes.htm). Every participant has given their written consent for the NHANES study, and the project has also received ethical approval (25). Therefore, this study did not necessitate any specific approval or ethical review.

### 2.2. Covariates

Several variables were considered to be associated with BRI and CKD based on other studies (26, 27). These include age, sex, race (mexican, other race, non-hispanic white, or non-hispanic black), education level (less than high school graduate, high school graduates, above high school, or not record), marital status (married, separated, or not record), poverty income ratio (<1.30, 1.30–3.50, ≥3.5, or not record), smoking status (never smoker, former smoker, current smoker, or not record), alcohol consumption (never, former, mild to moderate, heavy, or not record), bmi, hypertension (yes, no), diabetes (yes, no), hyperlipidemia (yes, no), and cardiovascular disease (CVD) (yes, no).

### 2.3 Measurement of BRI

The calculation for BRI was as follows (19):


$$\begin{array}{l} \text{BRI}=364.2\;-365.5\times \sqrt{1-\left(\frac{{\left(\frac{\text{wc}}{2\Pi }\right)}^{2}}{{\left(0.5\text{ height}\right)}^{2}}\right)} \end{array}$$

### 2.4 Assessment of CKD

CKD is defined as an estimated glomerular filtration rate (eGFR) <60 mL/min/1.73 m^2^ and/or urinary albumin to creatinine ratio (UACR) ≥30 mg/g (28, 29). The eGFR was computed through the CKD-Epidemiology Collaboration equation, which includes serum creatinine, age, sex, and ethnicity (30). The calculation for eGFR was as follows: 141 × min (SCr/κ, 1)^α^ × max (SCr/κ, 1)^−1.209^ × 0.993^Age^ × 1.018 if female × 1.159 if black, where SCr represents serum creatinine, κ is 0.7 for females and 0.9 for males, α is −0.329 for females and −0.411 for males, min denotes the lesser value between SCr/κ or 1, and max signifies the greater value between SCr/κ or 1.

### 2.5 Statistical analyses

Weighted analyses were carried out by the NHANES recommendations due to the complex sampling survey. Continuous variables are expressed as weighted median (IQR) and were compared using weighted linear regression analysis. Categorical variables are expressed as unweighted frequencies (weighted percentages) and were compared using the chi-squared test. Multivariate logistic regression analysis was used to evaluate the correlation between BRI and CKD in different models. Model I: confounding variables were not adjusted. Model II: age, sex, race, marital status, education levels family income-poverty ratio (PIR), smoking status, alcohol consumption, and bmi were adjusted. Model III: age, sex, race, education level, marital status, poverty income ratio (PIR), smoking status, alcohol consumption, bmi, hypertension, diabetes, hyperlipidemia, and cardiovascular disease (CVD). In addition, we use weighted restricted cubic splines (RCS) in Model III to assess the non-linear relationship between BRI and CKD. The receiver operating characteristic (ROC) curve and area under the curve (AUC) were used to evaluate the effectiveness of the variables BRI, a body shape index (ABSI), BMI, Body Weight, and waist circumstance (WC) in predicting CKD. Stratified analyses were performed in sex (female, male), hypertension (yes, no), diabetes (yes, no), hyperlipidemia (yes, no), and CVD (yes, no) to evaluate potential interactions between BRI and CKD.

All analyses were performed using R software version 4.2.2. Statistical significance was defined as two-sided *P* < 0.05.

## 3 Results

### 3.1 Characteristics of study participants at the baseline

**Figure 1:**
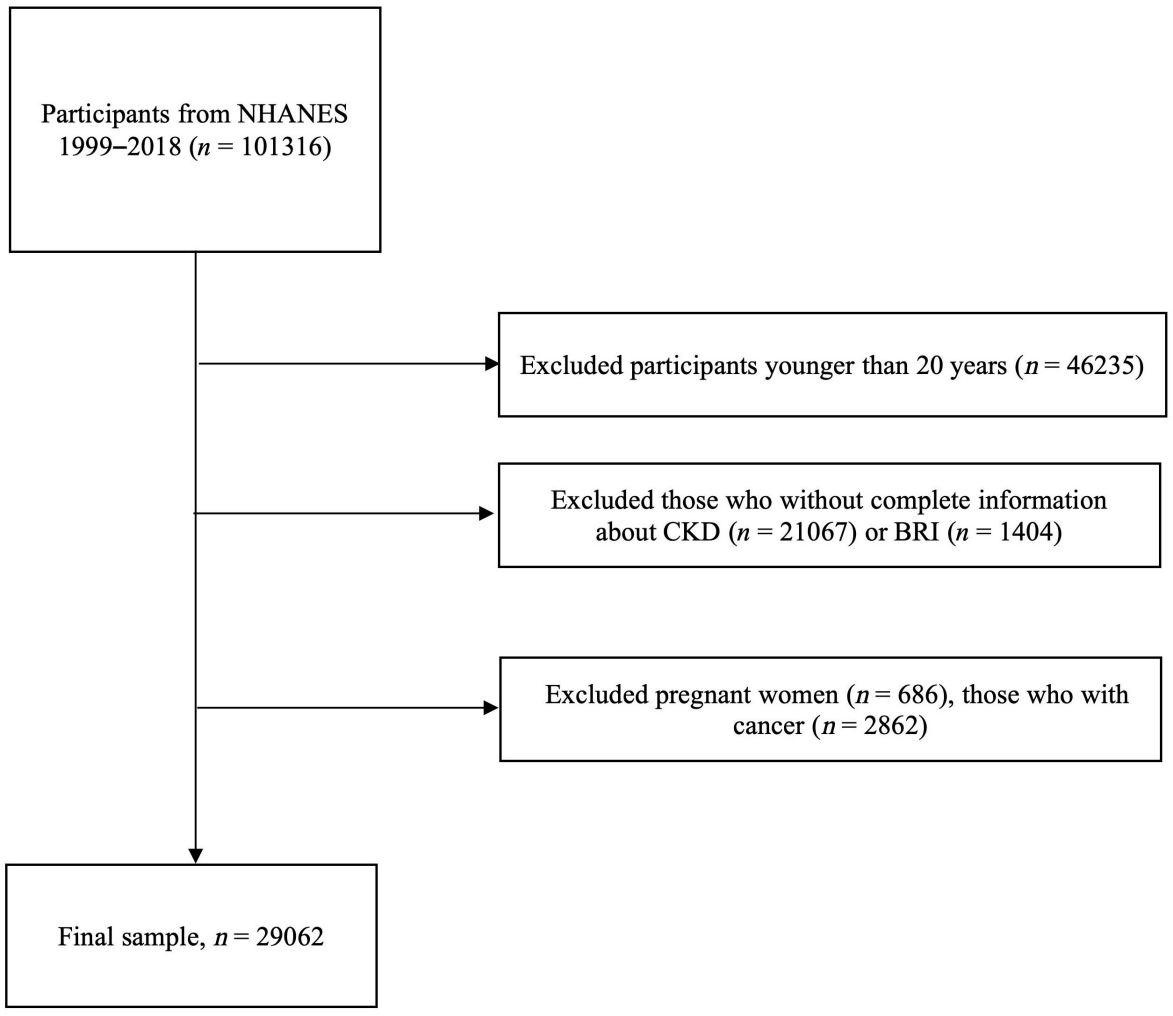
Flow chart of study participants.

There were a total of 101,316 participants from NHANES 1999–2018. Among them, participants younger than 20 years (*n* = 46,235), those with incomplete information about CKD (*n* = 21,067) or BRI (*n* = 1,404), pregnant women (*n* = 686), those with cancer (*n* = 2,862) were excluded. In total, 29,062 participants with complete infomation about BRI and CKD were enrolled in further analyses (Figure 1).

As shown in Table 1, The BRI was divided into four groups, namely, 2.50–3.33, 4.00–4.59, 5.24–5.99, and 7.07–9.24 in Q1, Q2, Q3, and Q4, respectively. The average age of the study population was 44.00 (32.00–57.00) years old, and 50.22% were female. The prevalence of CKD was reported in 12.30% of the participant and accounted for 6.97%, 9.20%, 13.42%, and 20.09% in groups Q1, Q2, Q3, and Q4, respectively. Individuals in Q4 had a higher level of BMI, HbA1c, UACR, and Serum uric acid, with higher prevalence of hypertension, diabetes, hyperlipidemia, and CVD (all *P* < 0.001). Further more, with the increase of the BRI, there was a signaificant increase in the prevalence of CKD (*P* < 0.001).

**Table 1:** Baseline characteristics.

**Characteristic**	**Body Roundness Index (BRI)**					***P***
	**Overall**	**Quantile 1**	**Quantile 2**	**Quantile 3**	**Quantile 4**	
	4.87 (3.64, 6.43)	2.94 (2.50, 3.33)	4.30 (4.00, 4.59)	5.59 (5.24, 5.99)	7.88 (7.07, 9.24)	
Participants, *n*	29,062	6,639	7,028	7,616	7,779	
Age, year	44.00 (32.00, 57.00)	34.00 (26.00, 47.00)	44.00 (33.00, 56.00)	49.00 (37.00, 61.00)	50.00 (37.00, 62.00)	< 0.001
**Sex**						
Female	14,620 (50.22%)	3,062 (49.34%)	3,105 (44.94%)	3,570 (46.03%)	4,883 (60.88%)	< 0.001
Male	14,442 (49.78%)	3,577 (50.66%)	3,923 (55.06%)	4,046 (53.97%)	2,896 (39.12%)	
**Race**						
Mexican	5,229 (8.88%)	620 (4.90%)	1,185 (8.46%)	1,755 (11.50%)	1,669 (10.92%)	< 0.001
Other Race	6,248 (14.27%)	1,661 (15.42%)	1,780 (16.43%)	1,567 (13.80%)	1,240 (11.27%)	
Non-Hispanic White	11,522 (65.78%)	2,865 (68.32%)	2,770 (65.81%)	2,860 (64.66%)	3,027 (64.17%)	
Non-Hispanic Black	6,063 (11.07%)	1,493 (11.35%)	1,293 (9.30%)	1,434 (10.04%)	1,843 (13.64%)	
**Education level**						
Less than high school	7,562 (16.56%)	1,186 (12.27%)	1,735 (15.64%)	2,293 (19.34%)	2,348 (19.28%)	< 0.001
High school graduates	6,607 (23.75%)	1,406 (20.34%)	1,532 (22.40%)	1,748 (24.97%)	1,921 (27.55%)	
Above high school	14,862 (59.62%)	4,039 (67.28%)	3,752 (61.88%)	3,566 (55.60%)	3,505 (53.15%)	
Not record	31 (0.08%)	8 (0.12%)	9 (0.08%)	9 (0.09%)	5 (0.02%)	
**Marital status**						
Married	14,883 (54.29%)	2,828 (45.51%)	3,929 (58.82%)	4,298 (60.11%)	3,828 (53.11%)	< 0.001
Separated	13,795 (44.09%)	3,711 (52.28%)	3,000 (39.23%)	3,231 (38.90%)	3,853 (45.63%)	
Not record	384 (1.61%)	100 (2.20%)	99 (1.96%)	87 (0.99%)	98 (1.26%)	
**Family income-to-poverty ratio**						
< 1.3	8,503 (20.66%)	1,818 (19.94%)	1,890 (18.88%)	2,177 (19.63%)	2,618 (24.32%)	< 0.001
1.3–3.5	9,887 (33.01%)	2,151 (30.76%)	2,308 (31.65%)	2,635 (33.36%)	2,793 (36.48%)	
≥3.5	7,957 (38.81%)	2,101 (41.69%)	2,176 (42.26%)	2,029 (39.09%)	1,651 (31.89%)	
Not record	2,715 (7.51%)	569 (7.60%)	654 (7.21%)	775 (7.92%)	717 (7.32%)	
BMI, kg/m^2^	27.80 (24.10, 32.30)	22.30 (20.61, 23.97)	26.20 (24.64, 27.87)	29.77 (28.00, 31.80)	36.20 (33.30, 40.30)	< 0.001
**BMI**						
< 25	8,523 (30.60%)	5,770 (86.34%)	2,396 (29.30%)	353 (3.24%)	4 (0.04%)	< 0.001
25–30	155 (0.30%)	0 (0%)	9 (0.04%)	58 (0.43%)	88 (0.75%)	
>30	20,384 (69.11%)	869 (13.66%)	4,623 (70.65%)	7,205 (86.34%)	7,687 (89.21%)	
WC, m	0.97 (0.87, 1.08)	0.81 (0.76, 0.85)	0.93 (0.89, 0.97)	1.03 (0.98, 1.07)	1.17 (1.10, 1.26)	< 0.001
**Smoking status**						
Never smoker	16,282 (55.41%)	3,849 (58.21%)	3,948 (55.65%)	4,146 (53.97%)	4,339 (53.65%)	< 0.001
Former smoker	6,678 (23.44%)	1,000 (16.67%)	1,568 (22.33%)	2,056 (26.83%)	2,054 (28.39%)	
Current smoker	6,084 (21.09%)	1,785 (25.06%)	1,507 (21.97%)	1,410 (19.16%)	1,382 (17.91%)	
Not record	18 (0.05%)	5 (0.06%)	5 (0.05%)	4 (0.05%)	4 (0.05%)	
**Alcohol consumption**						
Never	2,865 (7.48%)	531 (5.99%)	617 (6.61%)	781 (8.10%)	936 (9.35%)	< 0.001
Former	2,654 (7.60%)	480 (5.39%)	586 (7.02%)	683 (7.83%)	905 (10.32%)	
Mild to moderate	14,479 (52.54%)	3,502 (55.47%)	3,577 (51.27%)	3,742 (50.51%)	3,658 (48.66%)	
eavy	2,815 (11.74%)	810 (13.46%)	791 (14.42%)	754 (12.12%)	460 (6.76%)	
Not record	6,249 (21.63%)	1,316 (19.68%)	1,457 (20.68%)	1,656 (21.44%)	1,820 (24.91%)	
eGFR	99.00 (84.17, 113.04)	105.46 (91.40, 118.29)	98.25 (83.84, 112.00)	95.37 (81.21, 109.23)	96.33 (79.77, 110.59)	< 0.001
UACR, mg/g	6.23 (4.17, 11.07)	5.76 (4.01, 9.60)	5.65 (3.89, 9.62)	6.29 (4.22, 10.92)	7.73 (4.84, 15.72)	< 0.001
HbA1c, %	5.40 (5.10, 5.70)	5.20 (5.00, 5.40)	5.30 (5.10, 5.60)	5.50 (5.20, 5.80)	5.60 (5.40, 6.10)	< 0.001
Serum uric acid, mg/dL	5.30 (4.40, 6.30)	4.80 (4.00, 5.70)	5.20 (4.30, 6.20)	5.50 (4.60, 6.50)	5.70 (4.80, 6.80)	< 0.001
Hypertension	11,703 (34.68%)	1,187 (14.55%)	2,391 (29.11%)	3,505 (40.80%)	4,620 (55.76%)	< 0.001
Diabetes	4,587 (11.23%)	222 (2.12%)	704 (6.07%)	1,309 (12.42%)	2,352 (25.12%)	< 0.001
Hyperlipidemia	15,191 (50.52%)	1,991 (29.35%)	3,629 (50.43%)	4,646 (60.48%)	4,925 (63.18%)	< 0.001
CKD	4,623 (12.30%)	556 (6.97%)	891 (9.20%)	1,329 (13.42%)	1,847 (20.09%)	< 0.001
CVD	2,700 (7.16%)	266 (2.95%)	507 (5.46%)	828 (8.42%)	1,099 (12.12%)	< 0.001

Values are weighted median (IQR) for continuous variables or numbers (weighted %) for categorical variables.

BMI, body mass index; BRI, body roundess index; HbA1c, glycosylated hemoglobin; eGFR, estimated glomerular filtration rate; CKD, chronic kidney disease; CVD, cardiovascular disease.

### 3.2 Associations between BRI and CKD

As shown in Table 2, multiple logistic regression models study the relationship between BRI and CKD. Compared with the Q1 group, Q2, Q3, and Q4 in Model I had increased risk of CKD, and the OR (95% CI) values were 1.35 (1.14, 1.60), 2.07 (1.79, 2.39), and 3.35 (2.94, 3.83), respectively. After adjusting for age, sex, race, marital status, education levels and family income-poverty ratio, bmi, smoking status, and alcohol consumption, the OR (95% CI) values in Model II were 0.88 (0.73, 1.05), 1.11 (0.93, 1.32), and 1.63 (1.32, 2.01) compared with the Q1 group, respectively. After further adjustment for hypertension, hyperlipidemia, diabetes, and cvd, the OR (95% CI) in Model III were 0.88 (0.73, 1.06), 1.05 (0.87, 1.26), and 1.36 (1.10, 1.70) compared with the Q1 group, respectively. In addition, we convert BRI from a categorical variable to a continuous variable for further analysis. This association is significant in our Model I (OR: 1.02; 95% CI 1.01, 1.02, *P* < 0.001), Model II (OR: 1.25; 95% CI 1.19, 1.32, *P* < 0.001), and Model III (OR: 1.16, 95% CI 1.10, 1.23, *P* < 0.001). However, as shown in Table 3, when stratified by race, the correlation was not significant for Mexican participants (OR: 1.10, 95% CI: 0.98–1.23). So we excluded Mexican participants for BMI stratification analysis, we found that there was a significant correlation between BRI and the risk of CKD for participants who were overweight (OR: 1.03, 95% CI: 1.02–1.06) and obese (OR: 1.10, 95% CI: 1.07–1.14). Whatsmore, the consistent correlation was observed in all participants.

**Table 2:** The relationship between BRI and CKD in NHANES 1999–2018.

	**Model I**	**Model II**	**Model III**
Continuous BRI	1.02 (1.01, 1.02)	1.25 (1.19, 1.32)	1.16 (1.10, 1.23)
*P* value	< 0.001	< 0.001	< 0.001
**Stratified by BRI**			
Q1 2.94 (2.50, 3.33)	Reference	Reference	Reference
Q2 4.30 (4.00, 4.59)	1.35 (1.14, 1.60)	0.88 (0.73, 1.05)	0.88 (0.73, 1.06)
Q3 5.59 (5.24, 5.99)	2.07 (1.79, 2.39)	1.11 (0.93, 1.32)	1.05 (0.87, 1.26)
Q4 7.88 (7.07, 9.24)	1.05 (0.87, 1.26)	1.63 (1.32, 2.01)	1.36 (1.10, 1.70)
*P* for trend	< 0.001	< 0.001	0.002

Values are adjusted OR (95% CI). Model I was the crude model. Model II adjusted for age, sex, race, marital status, education levels and family income-poverty ratio, BMI, smoking status, alcohol consumption. Model III adjusted for age, sex, race, education levels and family income-poverty ratio, BMI, smoking status, alcohol consumption, hypertension, hyperlipidemia, diabetes, and CVD.

**Table 3:** The relationship between BRI and CKD in NHANES 1999–2018 by race and BMI.

	**Model I**	**Model II**	**Model III**
**Stratified by race**			
Mexican	1.02 (1.01, 1.02)	1.14 (1.02, 1.28)	1.10 (0.98, 1.23)
Other race	1.03 (1.02, 1.04)	1.26 (1.10, 1.45)	1.16 (1.01, 1.34)
Non-Hispanic White	1.02 (1.02, 1.03)	1.27 (1.17, 1.37)	1.17 (1.08, 1.27)
Non-Hispanic Black	1.02 (1.01, 1.02)	1.29 (1.17, 1.41)	1.20 (1.08, 1.33)
**Stratified by BMI excluding Mexican participants**			
Under/normal weight	1.05 (1.04, 1.07)	1.01 (0.89, 1.14)	0.92 (0.81, 1.05)
Overweight	1.01 (1.01, 1.02)	1.04 (1.03, 1.07)	1.03 (1.02, 1.06)
Obese	1.03 (1.02, 1.03)	1.17 (1.14, 1.21)	1.10 (1.07, 1.14)
**Stratified by BMI in all participants**			
Under/normal weight	1.05 (1.04, 1.06)	1.01 (0.90, 1.15)	0.93 (0.82, 1.05)
Overweight	1.01 (1.01, 1.02)	1.04 (1.03, 1.08)	1.02 (1.01, 1.06)
Obese	1.03 (1.02, 1.03)	1.17 (1.14, 1.20)	1.09 (1.06, 1.13)

Values are adjusted OR (95% CI). Model I was the crude model. Model II adjusted for age, sex, race, marital status, education levels and family income-poverty ratio, BMI, smoking status, alcohol consumption. Model III adjusted for age, sex, race, education levels and family income-poverty ratio, BMI, smoking status, alcohol consumption, hypertension, hyperlipidemia, diabetes, and CVD.

In the subgroup analysis by Race and BMI, the model was not adjusted for the stratification variable itself.

**Figure 2:**
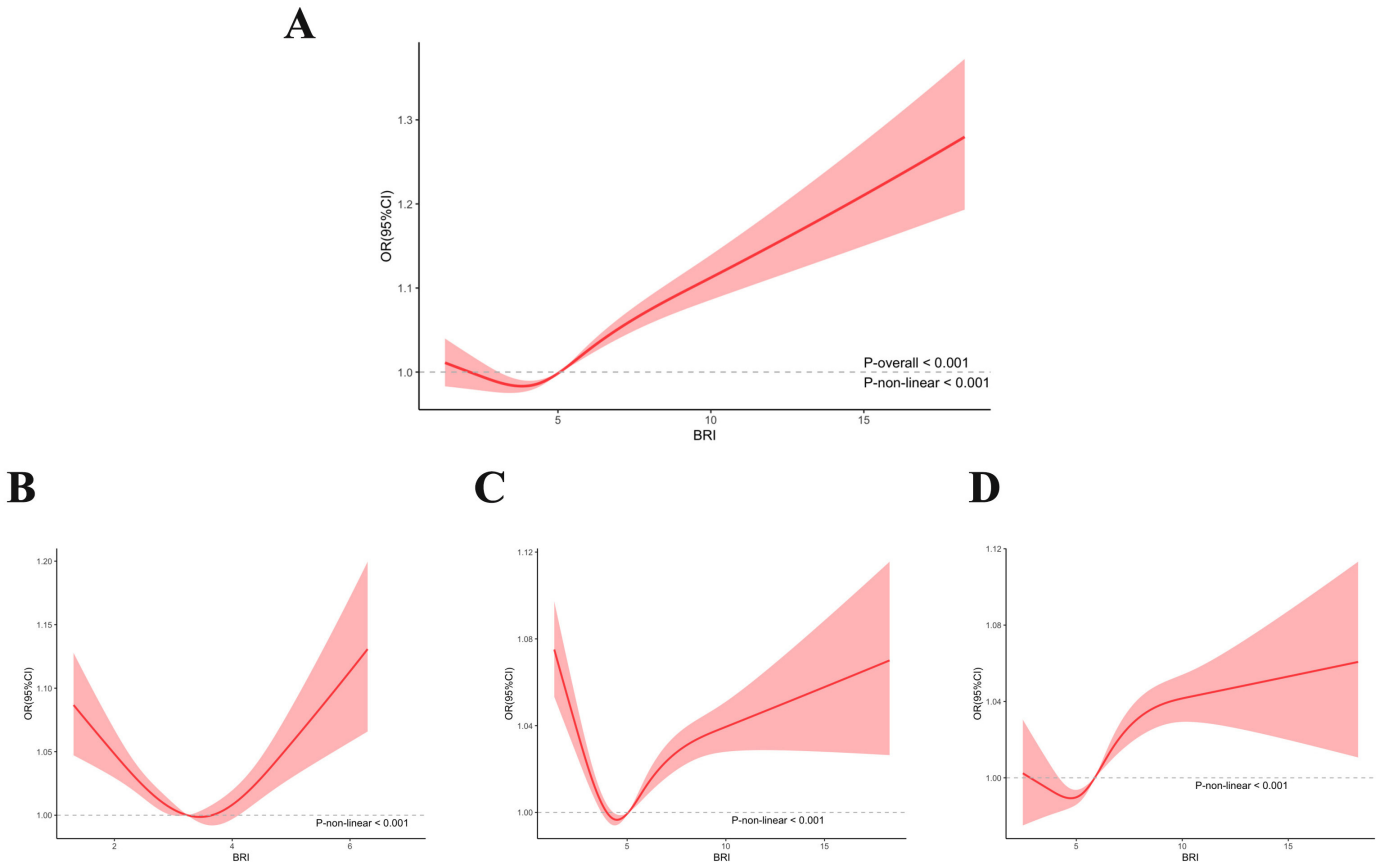
Non-linear association between BRI and CKD by the restricted spline model in all participants **(A)** under/normal weight participants **(B)** overweight participants **(C)**, and obese participants **(D)**.

RCS regression with multivariable-adjusted associations was used to demonstrate associations between BRI and the prevalence of CKD. The RCS curve revealed a U-shaped nonlinear correlation with the prevalence of CKD in all participants (Figure 2A). Even when we conducted a stratified analysis based on BMI, the nonlinear correlation still existed (Figures 2B–D) (*P* for nonlinearity < 0.001).

### 3.3. BRI had the best diagnostic ablility to predict CKD

**Figure 3:**
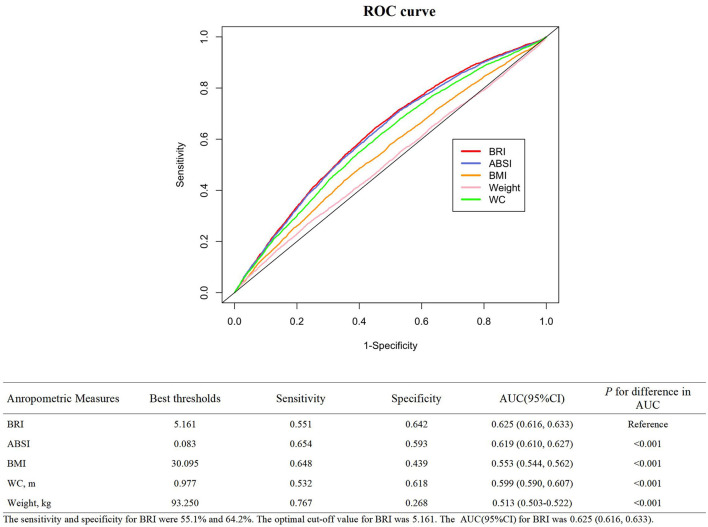
ROC curves of different anthropometric indices for the prediction of CKD risk.

As shown in Figure 3, ROC curves were plotted to investigate the abilities of five anthropometric indexes in discriminating individuals with CKD. The corresponding sensitivity and specificity were 55.1% and 64.2% and the optimal cut-off value for BRI was 5.161. Whatsmore, compared to ABSI (AUC: 0.619 95% CI 0.610, 0.627), BMI (AUC: 0.553 95% CI 0.544, 0.562), WC (AUC: 0.599 95% CI 0.590, 0.607), and body weight (AUC: 0.513 95% CI 0.503, 0.522), BRI (AUC: 0.625; 95% CI 0.616, 0.633, all *P* < 0.001) had the strongest diagnostic capacity in our study.

### 3.4 Subgroup analyses for the association between BRI and CKD

**Figure 4:**
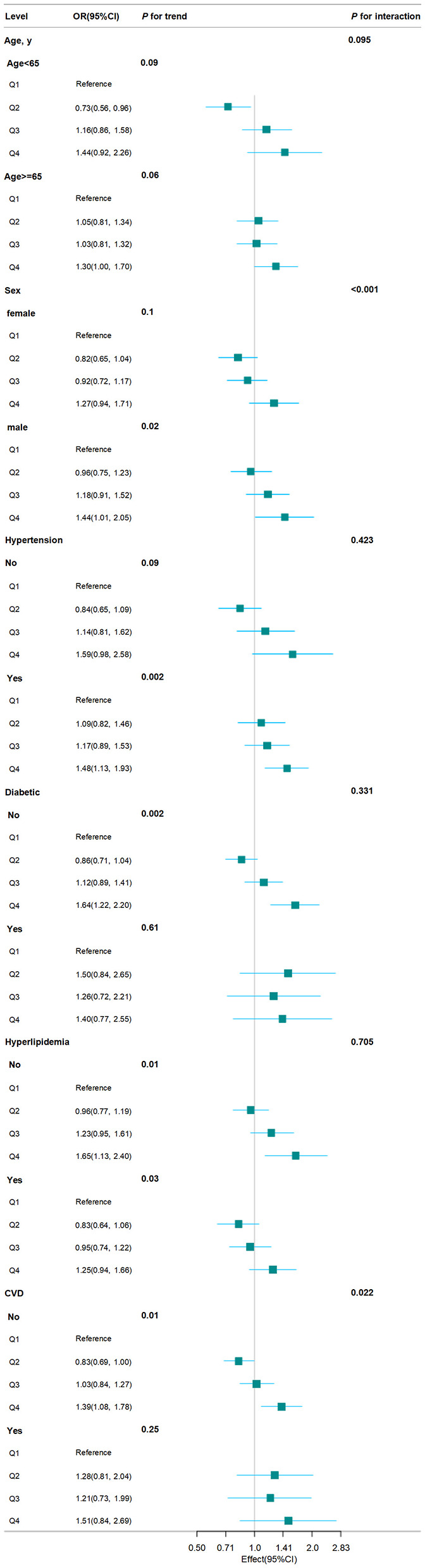
Subgroup analysis for association between BRI and CKD.

We conducted stratified analyses to investigate whether subgroups affected the relationship between BRI and CKD prevalence. As shown in Figure 4, the stratified analysis indicated that participants who were male or had no history of diabetes, hyperlipidemia, and CVD exhibited a higher risk of CKD in the Q4 of BRI compared to those in the Q1 (all *P* for trend < 0.05). According to the interaction test, hypertension, diabetes, and hyperlipidemia had no significant impact on the positive connection between BRI and CKD (all *P* for interaction > 0.05). Contrary, sex and CVD may influence the positive association between BRI and CKD (all *P* for interaction < 0.05).

## 4 Discussion

In this large-scale cross-sectional study, which included 29,062 participants, we found a strong association between BRI and CKD. Dose–response analysis of BRI and CKD showed a nonlinear positive correlation between BRI and CKD. This correlation persisted even after controlling for confounding factors, suggesting that CKD could be impacted by BRI. For every unit increase in BRI, the probability of CKD rose by 16%. In addition, BRI has the best diagnostic ablility to predict the risk of CKD than ABSI, BMI, WC, and Body Weight, as indicated by the largest AUC. Subgroup analysis showed that individuals with a higher BRI showed a higher risk of CKD, particularly among those who without diabetes, hyperlipidemia, and CVD. Our findings revealed that higher BRI were an independently risk factor for CKD.

Obesity is an increasingly serious global epidemic, especially in developed countries like the US where the obese population continues to increase (31). Obesity is a representative risk factor for the development and progression of chronic kidney disease to end-stage renal disease (5–7). Hemodynamic abnormalities, metabolic disorders, lipid toxicity, and inflammatory response collectively contribute to the development and progression of CKD in obese patients. Our study found a U-shaped relationship between BRI and the risk of CKD, which seems reasonable as BRI has been identified as a potential alternative indicator related to nutritional status. Extremely low BRI may be associated with malnutrition, fatigue, reduced activity tolerance, and muscle atrophy, leading to an increased risk of CKD (32, 33). When BRI exceeds 5.161, we observed a significant positive correlation with CKD incidence. Moreover, our subgroup analysis suggests that sex and CVD history may affect the positive association between BRI and CKD. On one hand, the influence of sex hormones on body composition and fat distribution may result in men having a greater risk of developing CKD compared to women (34). On the other hand, individuals with a history of CVD might already experience compromised vascular health (35), potentially leading to a weaker association between BRI and CKD in the CVD population compared to those without a history of CVD. Therefore, maintaining an appropriate BRI value is very important for health.

Compared to conventional body measurement indicators, BRI has the benefit of precisely estimating the percentage of visceral adipose tissue and body fat, which can better reflect the distribution of fat. By evaluating obesity risk based on waist circumference, height, and body roundness, the BRI provides a deeper assessment of fat distribution patterns. Therefore, it can offer greater accuracy in assessing the risk of chronic kidney disease, cardiovascular diseases, and diabetes compared to traditional anthropometric measures such as BMI and WC. Whatsmore, ABSI, as a novel obesity measurement index, is mainly used to evaluate the impact of waist fat on health status (36). Numerous studies have demonstrated that BRI exhibits a stronger predictive ability for metabolic disorders compared to ABSI. For example, a study by Anto et al., (37). revealed that after adjusting for all variables, the odds ratio of ABSI in relation to metabolic syndrome risk was not statistically significant, whereas BRI remained significant. Similarly, Li et al. (38). highlighted BRI's effective, non-invasive nature in predicting conditions such as hypertension and hyperuricemia, surpassing the predictive power of ABSI. We have also come to the same conclusion in our research: BRI has the best diagnostic ablility to predict the risk of CKD than ABSI, BMI, WC, and Body Weight. Naturally, to thoroughly assess the diagnostic capabilities of both BRI and ABSI in CKD, more extensive research is required. Nonetheless, our study provides valuable insights and directions for further exploration in this field.

Our research found that the correlation between BRI and CKD is more pronounced in overweight and obese patients, which may enhance our understanding of BRI and help clinical doctors guide overweight and obese patients to help them control their body weight and reduce abdominal fat, ultimately lowering the risk of CKD. The most common causes of CKD are diabetes, hypertension and glomerulonephritis (39). Calderón-García et al.'s (40) meta-analysis revealed a substantial correlation between BRI and hypertension. Wu et al. (41) found an independent correlation between elevated baseline BRI levels and T2DM events. Zhang et al. (42) found that a U-shape association between BRI and all-cause mortality. To our knowledge, there is currently no research on the risk relationship between BRI and CKD in US adults. Our research demonstrates that BRI has a strong correlation with the risk of CKD in overweight and obese patients.

The study has several limitations. First, due to its cross-sectional design, it is impossible to determine a causal relationship between BRI and the occurrence of CKD. Second, even after adjusting for potential confounding factors, residual confounding factors may still exist and alter the connection between the BRI and CKD. Thirdly, we did not accurately classify obesity based on BMI classification requirements for different races, which may cause some interference with the results of stratified analysis. Finally, the NHANES database adopted a multistage sampling method with a weighting scheme to ensure a large and representative sample size. However, due to the excluded individuals under the age of 20, pregnant women, and cancer patients from our study, the results cannot be generalized to all populations. Therefore, more extensive and comprehensive research is needed to further elucidate the association between BRI and the risk of CKD in all populations.

## 5 Conclusion

BRI was independently associated with a higher prevalence of CKD in overweight and obese US adults, excluding Mexican. This may be an important therapeutic target and predictor of CKD. Physicians should advise patients with high BRI scores, especially overweight and obese patients, to embrace healthy lifestyle changes, such as maintaining a balanced diet and engaging in regular physical activity. These changes can help them control their body weight and reduce abdominal fat, ultimately lowering the risk of CKD.

## Data Availability

The raw data supporting the conclusions of this article will be made available by the authors, without undue reservation.
